# High Temperature Behaviors of a Casting Nickel-Based Superalloy Used for 815 °C

**DOI:** 10.3390/ma14040716

**Published:** 2021-02-04

**Authors:** Jiangping Yu, Donghong Wang, Jingyang Chen, Changlin Yang, Xin Hao, Jianxin Zhou, Dayong Li, Da Shu, Chengbo Xiao, Yinghong Peng

**Affiliations:** 1 Shanghai Key Laboratory of Advanced High Temperature Materials and Precision Forming, School of Materials Science and Engineering, Shanghai Jiao Tong University, Shanghai 200240, China; jumpingyu@sjtu.edu.cn (J.Y.); dshu@sjtu.edu.cn (D.S.); 2 State Key Laboratory of Metal Matrix Composites, School of Materials Science and Engineering, Shanghai Jiao Tong University, Shanghai 200240, China; 3 State Key Laboratory of Mechanical System and Vibration, School of Mechanical Engineering, Shanghai Jiao Tong University, Shanghai 200240, China; dyli@sjtu.edu.cn (D.L.); yhpeng@sjtu.edu.cn (Y.P.); 4 Science and Technology on Advanced High Temperature Structural Materials Laboratory, Beijing Institute of Aeronautical Materials, Beijing 100095, China; jychen126@126.com (J.C.); cbxiao0288@sina.com (C.X.); 5 State Key Laboratory of Solidification Processing, Northwestern Polytechnical University, Xi’an 710072, China; ycl@nwpu.edu.cn (C.Y.); haoxin_csu@163.com (X.H.); 6 State Key Laboratory of Material Processing and Die & Mould Technology, Huazhong University of Science and Technology, Wuhan 430074, China; zhoujianxin@hust.edu.cn

**Keywords:** nickel-based superalloy, high-temperature deformation, investment casting, hot compression test, mechanical properties

## Abstract

The hot deformation behaviors of the SJTU-1 alloy, the high-throughput scanned casting Nickel-based superalloy, was investigated by compression test in the temperature range of 900 to 1200 °C and strain rate range of 0.1–0.001 s^−1^. The hot processing map has been constructed with the instability zone. At the beginning of hot deformation, the flow stress moves rapidly to the peak value with the increased strain rates. Meanwhile, the peak stress is decreased with the increased temperature at the same strain rates. However, the peak stress shows the same tendency with the strain rates at the same temperature. The optimum hot deformation condition was determined in the temperature range of 1000–1075 °C, and the strain rate range of 0.005–0.1 s^−1^. The microstructure investigation indicates the strain rate significantly affects the characteristics of the microstructure. The deformation constitutive equation has also been discussed as well.

## 1. Introduction

Nickel-based superalloy is widely used for crucial structural parts of rock engine, turbine blades, cartridge receiver, and other components bearing high-temperature loads for decades due to its outstanding mechanical properties and extraordinary oxidation resistance at temperatures close to 650 °C [1,2,3,4]. Meanwhile, it is difficult to get a shape transformation due to its stubborn deformation resistance such as high strength, weak thermal diffusivity as well as work hardening behavior at elevated temperature. For the promotion of the fuel efficiency of such significant components, it is urgent to develop novel Ni-based superalloys to sustain hostile environment attacks such as high temperature and high pressure, thus significantly reducing the service life. For the lower cost and higher benefit of the machining and forming, it is in desperate need of a commercial cast-wrought superalloy to withstand high-temperature loads as well as high cyclic loading. SJTU-1 alloy, known as Ni-based superalloy and excavated from 5.2 million components after high throughput component design, was developed because of its exceptional high-temperature strength, toughness, and outstanding resistance to degradation in corrosive or oxidizing environments. Based on the existing composition and properties of the casting alloy, a Nickel-based superalloy named SJTU-1 alloy was excavated from high throughput component design with its exceptional high-temperature strength, good toughness and outstanding resistance to degradation in corrosive or oxidizing environments.

As is known, hot deformation behavior is vital for manufacture when carrying mental production into reality and it is also affected by many factors [5,6]. The mechanism of hot deformation and recrystallization mechanism of different superalloy varies much though the constitutive equations are prevalent to describe the deformation characteristics [7,8,9]. The Arrhenius model is typical and fluently used to describe the relationship between the flow stress and deformation temperature and strain rate [10,11,12]. Recent progress in processing maps has enabled researchers to have a better understanding of the mechanism of the hot deformation behavior of a metallic material [13,14,15]. Zhang and Li [16] put an insight into the hot deformation behavior of In718 during isothermal compression deformation, it was concluded that the peak stress is decreased due to the absence of DRX. Lin et al. [17] studied the effects of WH, DRV, and DRX work hardening (WH), dynamic recrystallization (DRX) and dynamic recovery (DRV) on high-temperature deformation behaviors of a typical Ni-based superalloy and proposed an improved dislocation density-based model method to quantificationally describe the flow behaviors. Hence, giving a deeper insight into the hot deformation behavior is of great importance and in turn, guiding on the improved castability and moldability with optimized parameters.

Although the above mentioned constitutive models for Ni-based superalloys have aroused extensive interest and fully investigated, the novel high-throughput scanned superalloy has not been studied yet. In this study, a high-throughput scanning method was performed for the material design, and a novel superalloy named SJTU-1 alloy is obtained through an integrated thermodynamic computing platform. Then, the hot compression tests were carried out at different temperatures and strain rates as well. Moreover, the hot deformation constitutive equations, which are based on the modified Arrhenius model with a hyperbolic sine form coupled with the deformation activation energy and the temperature, is fully developed. Furthermore, the hot processing map is built and the optimum processing condition is confirmed. Last but not least, the microstructure of the compressed material is investigated to explore the effects of deformation temperature as well as strain rates on the metallic microstructure evolution.

## 2. Experimental

### 2.1. Materials Design and Its Characterization

For the basic performance in demand of a novel casting polycrystalline nickel-base superalloy, a Linux cluster computing platform has been built based on Thermo-Calc/DICTRA (Thermo-Calc Software AB, 2019a) with its corresponding thermodynamics and kinetics of nickel-based database. The thermodynamic parallel computing engine is aimed at the design and screening of high-throughput components, which can meet the demands of polycrystalline nickel-base superalloy casting development. The calculation platform was used to calculate more than 5.2 million components. In combination with the criteria of initial melting temperature, solidification temperature interval, and strengthening phase fraction, 22,398 component combinations were preliminarily screened. The relative creep rate, surface environmental resistance, and other criteria were further used to obtain 842 alternative systems. Finally, one basic composition combination (named at SJTU-1 alloy) was selected and it turned out that the creep properties of the alloys were better than or close to those of IN939 through JMatPro (Sente Software Ltd., Version 7.0.0) calculation and from Moattari [18] and Jahangiri [19].

**Figure 1:**
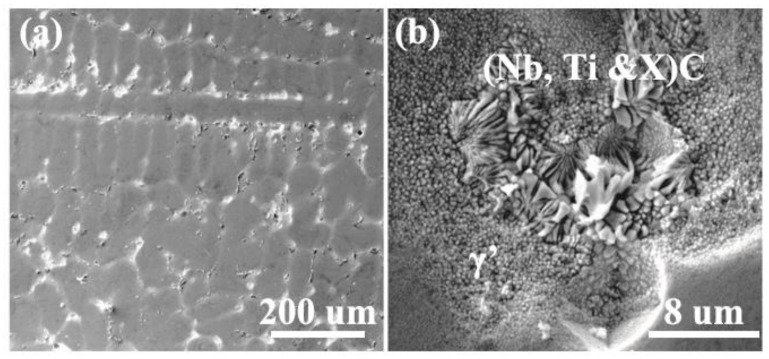
The microstructure of the SJTU-1 alloy in (**a**) OM and (**b**) SEM.

The sample adopted the self-developed casting Nickel-based superalloy SJTU-1 alloy, and the composition was tested and shown in Table 1 below. The initial microstructure of the as-received alloy is given in Figure 1. It demonstrated a typical dendritic structure with a large number of fine Gamma Prime and (Nb, Ti, X) C and other composite carbides between the dendrites.

**Figure 2:**
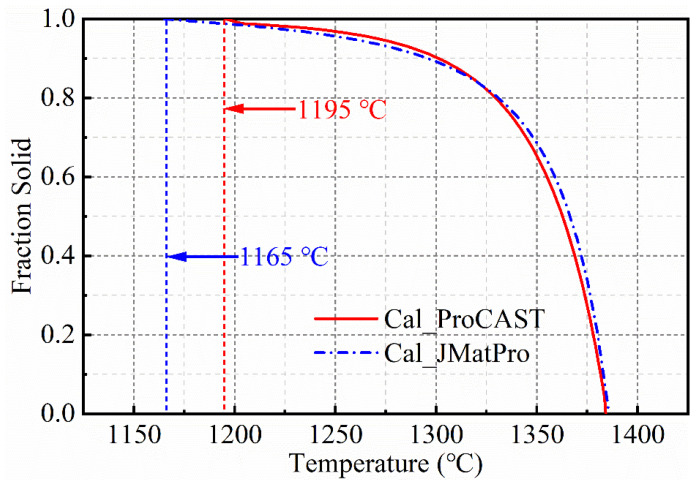
Fraction solid-temperature cure of SJTU-1 alloy calculated by ProCAST and JMatPro respectively.

The relationship between fraction liquid and temperature of SJTU-1 alloy calculated by ProCAST (ESI Group, Version 2019) and JMatPro is shown in Figure 2. It can be concluded that the minimum eutectic temperature (solidus temperature) is in the range of 1165–1195 °C, less than 1200 °C.

### 2.2. Tensile Test at 850 °C

The tensile test was performed with the help of FL4000GL (FULETEST, Shanghai, China) machine at 850 °C. The dimension of the sample is cut according to the standard [20].

### 2.3. Hot Compression Test

The thermal compression test was carried out on the Gleeble 3800 (Dynamic System Inc., New Jersey, NJ, USA) thermal simulator. According to the requirements of the thermal simulation test, the sample was prepared with a size of Φ 8 mm × 12 mm. The lubricant was applied to both ends of the cylinder specimen and graphite sheets with a thickness of 0.05 mm were affixed to minimize the contact friction between the head and the specimen during thermal compression deformation. After the setup was ready, the sample was heated to the deformation temperature (900 °C, 1000 °C, 1050 °C, 1100 °C, 1150 °C, 1200 °C) at 10 °C/s, and the temperature was then kept for 3 min to eliminate the temperature gradient inside the sample. Finally, isothermal compression was performed with the strain rate of 0.1 s^−1^, 0.01 s^−1^, 0.001 s^−1^ and the maximum deformation amount was 60%. After the compression was completed, all samples were immediately water quenched to retain their deformed structure. The deformed sample was cut in half along the axial direction. After metallographic inlay, grinding, and polishing, the microstructure examination was finally observed under a ZEISS metallographic microscope (Carl Zeiss, Anfragen, Germany). The corrosive agent used in the experiment was 5 g CuCl + 100 mL HCl + 100 mL C_2_H_5_OH.

## 3. Results and Discussion

### 3.1. Stress-Strain Curves

**Figure 3:**
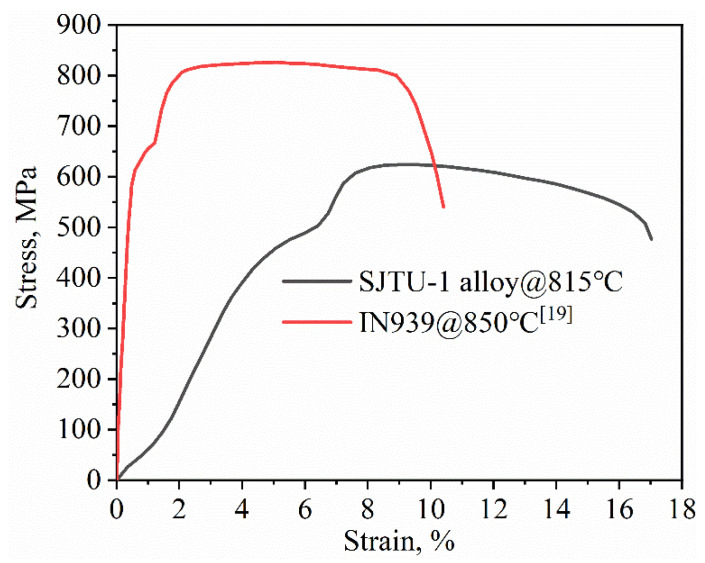
Comparison of the tensile property of SJTU-1 alloy and IN939.

The tensile property of SJTU-1 alloy used at 815 °C was performed and a further comparison was obtained from Figure 3. It turned out that the tensile strength is approximately 810 MPa and the elongation exceeds 10% at 850 °C, which is far overweight that of IN939 reported in [18,19]. Additionally, as the strain increased, the stress reached the yield point. Impressively, a sudden increase occurred, which was attributed to the irregular nucleation and propagation of deformation bands and the refinement of the coarse γ′ particles [21]. Hence, the SJTU-1 alloy with excellent properties is a promising alloy that is worthy of further investigation.

**Figure 4:**
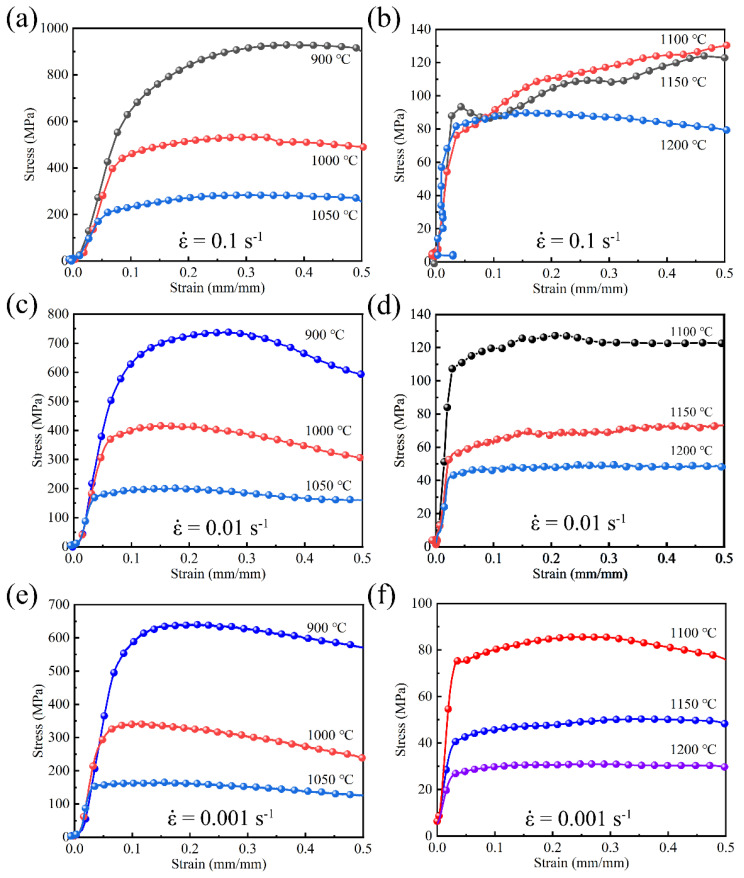
True stress-strain curves of SJTU-1 alloy at different strain rates: (**a**,**b**) at 0.1 s^−1^; (**c**,**d**) at 0.01 s^−1^; (**e**,**f**) at 0.001 s^−1^.

Figure 4 shows the true stress-strain curve of SJTU-1 alloy under isothermal thermal compression with different deformation conditions. It can be seen from Figure 4 that the flow stress increases rapidly as the deformation increases. While reaching a certain maximum value, it gradually tends to a steady-state or somehow decreases slightly. This is due to the dominance of work hardening in the initial stage of thermal deformation. With the continuous progress of thermal deformation, the change of flow stress begins to show different characteristics due to the difference in thermal deformation temperature and strain rate because the dynamic recrystallization hereafter plays a dominant role.

At the same strain rate, the flow stress decreases with the increase of the deformation temperature. However, the flow stress increases with the increase of the strain rate at the same deformation temperature. When the stress reaches the peak, the flow stress decreases gradually with the increase of strain. It may be ascribed to the dislocation accumulation. As the increased strain rate going up, the dislocation density is generated in a short time and the dislocation motion is severely blocked, resulting in the reduction of the softening rate caused by dislocation climbing and dislocation reaction. Therefore, the alloy is strengthened and the critical shear stress of the alloy increases, leading to an increase in flow stress.

It can also be seen from Figure 4 that the curve under some conditions has Zigzag rheological characteristics, which is attributed to the alternations of hardening caused by grain growth and hardening caused by the re-deformation of recrystallized grains in dynamic recrystallization. Apparently, work hardening predominates before peak stress. When the strain continues to increase, the dislocation density increases continuously, and the deformation storage can become the driving force of recrystallization after exceeding a certain value of deformation. The softening effect caused by dynamic recrystallization gradually plays a decisive role in the deformation process. When the work hardening rate and the dynamic softening rate came into balance, the flow stress finally reaches its peak value. With the progress of dynamic recrystallization, the softening rate was higher than the hardening rate, and the stress gradually decreased. In Figure 4b, the stress curves (0.1 s^−1^, at 1100 °C and 1150 °C) are extremely irregular at the strain rate of 0.1 s^−1^, which may be due to the occurrence of cracks and subsequent rapid recovery of the crack in a short time by continuing compression.

**Figure 5:**
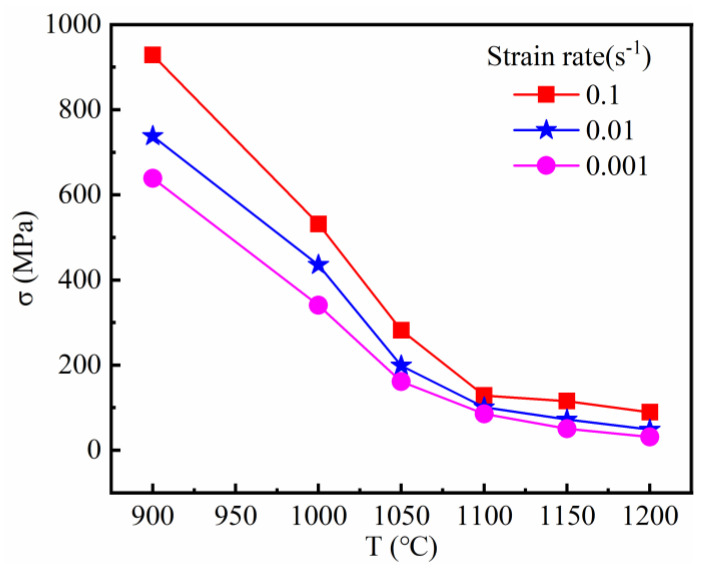
Relationship between peak stress and temperature and strain rate.

Moreover, the peak stress of the alloy decreases with the increase of temperature under a constant strain rate, as can be seen from Figure 5. The bonding force between atoms is getting weaker and weaker when the temperature is still increasing after the peak stress, promoting dislocation slip and grain boundary diffusion and sharpening the dislocation motion resistance affected by thermal activation. So the stress finally decreased.

### 3.2. The Rheological Constitutive Equation of Hot Compression

Hot deformation is a process that severely depends on thermal activation. In the process of high-temperature thermal deformation, flow stress (*σ*) is a very important factor affecting material processing, which is governed by the deformation temperature and strain rate. The peak stress is the largest in the stress-strain curve and can be chosen as the representative stress of each stress curve. Research on different thermal processes shows that *σ*, $\dot{\varepsilon }$, and *T* satisfy different relationships under different stress conditions. Under low-stress levels and high-stress levels, the relationship between flow stress and strain rate can be described by exponential relationship and power exponential relationship respectively.

At low-stress level (ασ < 0.8):

(1) $$\dot{\varepsilon }=A_{1}\sigma^{n_{1}}\exp[-Q/(RT)]$$

At high-stress levels (ασ > 1.2):

(2) $$\dot{\varepsilon }=A_{2}\exp(\beta \sigma )\exp[-Q/(RT)]$$

where σ is the flow stress (MPa), $\dot{\varepsilon }$ is the strain rate, *A*_1_(/s), *A*_2_(/s), *n*_1_(/s), *α*(MPa^−1^) and *β*(MPa^−1^) are the constants independent of temperature; *R* is the molar gas constant (8.3145 Jmol^−1^ °C^−1^); *T* is the deformation temperature; *Q* is the deformation activation energy (J/mol), which determines the difficulty of thermal deformation of the material, and is crucial for the process of thermal deformation. The above relationship (Equations (1) and (2)) describes the dynamic balance between strain hardening and dynamic softening. Considering the limitations of Equations (1) and (2), the modified Arrhenius relationship including the hyperbolic sine form of the deformation activation energy *Q* and the temperature *T* can be used to describe this thermally activated steady-state deformation behavior:

(3) $$\dot{\varepsilon }=A{\left[\sinh(\alpha \sigma )\right]}^{n}\exp[-Q/(RT)]$$

where *A* and *n* are all material constants, *α* is the stress exponent, and the relationship between *α*, *β*, and n_1_ is *α* = *β*/*n*_1_. Under low stress and high-stress conditions, Equation (3) can be simplified to Equations (1) and (2) respectively, so it can better describe the flow stress law of conventional thermal processing in the entire stress range. There is a thermal activation process for high-temperature plastic deformation. Furthermore, the effect of strain rate and temperature on flow stress can be expressed by the Zener-Hollomon (*Z*) parameter [22]:

(4) $$Z=\dot{\varepsilon }\exp[Q/(RT)]=A{\left[\sinh(\alpha \sigma )\right]}^{n}$$

*Z* is the deformation rate parameter of temperature compensation. From Equation (4),

(5) $$\sinh(\alpha \sigma )={\left(\frac{Z}{A}\right)}^{1/n}$$

According to the inverse function formula of the hyperbolic sine function:

(6) $$\mathrm{arcsinh}(\alpha \sigma )=\ln\left[\alpha \sigma +{\left(\alpha^{2}\sigma^{2}+1\right)}^{1/2}\right]$$

During the thermal deformation process, the flow stress *σ* of the superalloy mainly depends on the deformation temperature and strain rate, so the flow stress *σ* can be expressed as a function of the *Z* parameter, namely

(7) $$\sigma =\frac{1}{\alpha }\ln\left\{{\left(\frac{Z}{A}\right)}^{1/n}+{\left[{\left(\frac{Z}{A}\right)}^{2/n}+1\right]}^{1/2}\right\}$$

It can be seen from Equation (7) that if the material parameters such as *A*, *Q*, *n*, and *α* are known, the flow stress under arbitrary deformation conditions can be obtained. This study uses a hyperbolic sine function to describe the flow stress of SJTU-1 alloy. Assuming that the deformation activation energy *Q* is a constant at a certain temperature, the natural logarithms of both sides of Equations (1) and (2) are:

(8) $$\ln\dot{\varepsilon }=B_{1}+n_{1}\ln\sigma$$

(9) $$\ln\dot{\varepsilon }=B_{2}+\beta \sigma$$

where, $B_{1}=\ln A_{1}-Q/(RT)$, $B_{2}=\ln A_{2}-Q/(RT)$.

**Figure 6:**
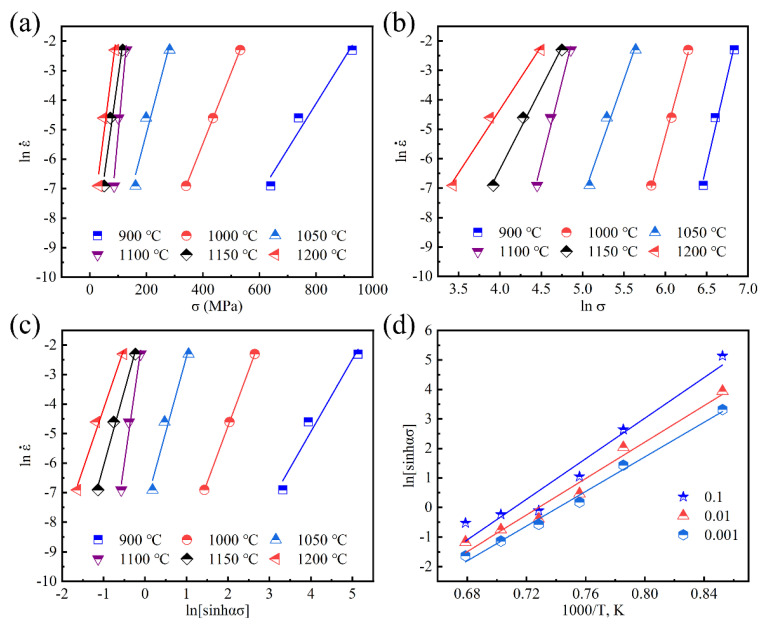
(**a**,**b**) Relationship between peak stress and strain rate; (**c**) Relationship between ln[sinh(*ασ*)] and ln$\dot{\varepsilon }$; (**d**) Relationship between ln[sinh(*ασ*)] and 1/*T*.

A large number of research results show that the peak stress and steady-state flow stress satisfy a certain linear relationship, and the peak stress and steady-state flow stress can be better described in the form of *Z* parameter functions. For industrial applications, it is more important to obtain the relationship between peak stress and temperature and strain rate. Take the peak stress under different conditions as the flow stress, different relationship curves can be drawn with a performed fitting linear regression respectively as shown in Figure 6. It can be seen from Figure 6a, b that the linear relationships with $\ln\dot{\varepsilon }$ and *σ*, $\ln\dot{\varepsilon }$ and ln*σ* are very obvious. According to Equations (8) and (9), the average value of the slopes of the straight lines in Figure 6a is obtained so *β* = 0.0538783 MPa^−1^. Likewise, from Figure 6b, *n*_1_ = 8.57393933. As a result, *α* = *β*/n_1_ = 0.00628 MPa^−1^.

Taking the natural logarithm on both sides of Equation (3), the following equation can be obtained.

(10) $$\ln\dot{\varepsilon }=B+n\ln[\sinh(\alpha \sigma )]$$

where, B=lnA−Q/(RT).

Using ln$\dot{\varepsilon }$ and ln[sinh(*ασ*)] as the coordinates to draw the corresponding graph with the help of *α* = 0.00628 MPa^−1^ and performing linear regression, the results are shown in Figure 6c. Impressively, the linear relationship between ln$\dot{\varepsilon }$ with ln[sinh(*ασ*)] is obvious, indicating that the relationship between flow stress and strain rate of SJTU-1 alloy can be well described by the Arrhenius relationship modified by a hyperbolic sine function.

Taking the natural logarithm of both sides of Equation (4), and assuming that when deforming under the condition of constant strain rate, *Q* remains stable within a certain temperature range, here we can get:

(11) $$\ln[\sinh(\alpha \sigma )]=A_{3}+B_{3}\frac{1000}{T}$$

where, $A_{3}=\frac{1}{n}(\ln\dot{\varepsilon }-\ln A)$;$B_{3}=\frac{Q}{1000nR}$.

Use ln[sinh(*ασ*)] and 1000/*T* as the coordinates to plot and then perform linear regression, as shown in Figure 6d. It can be seen that under the same strain rate, ln[sinh(*ασ*)] has a linear relationship with 1000/*T*. Considering the influence of temperature on the activation energy of deformation, the partial differentiation of Equation (4) gives:

(12) $$Q=R{\left\{\frac{\partial \ln\dot{\varepsilon }}{\partial \ln[\sinh(\alpha \sigma )]}\right\}}_{T}{\left\{\frac{\partial \ln[\sinh(\alpha \sigma )]}{\partial (1/T)}\right\}}_{\dot{\varepsilon }}=RnS$$

where *n* is the slope of the ln$\dot{\varepsilon }$-ln[sinh(*ασ*)] relationship at a certain temperature, i.e., the average of the slopes of the straight lines in Figure 6c, and its value is 5.0469. S is the slope of the ln[sinh(*ασ*)]-(1/*T*) relationship under the condition of a certain strain rate, i.e., the average value of the slope of each straight line in Figure 6d and its value is 31.525. Substituting *n* and *S* into Equation (12) can obtain deformation activation energy *Q* = 8.314 × 5.0469 × 31.525 = 1322.786 kJ/mol.

Taking the logarithm of both sides of Equation (4) can also get:

(13) lnZ=lnA+nln[sinh(ασ)]

**Figure 7:**
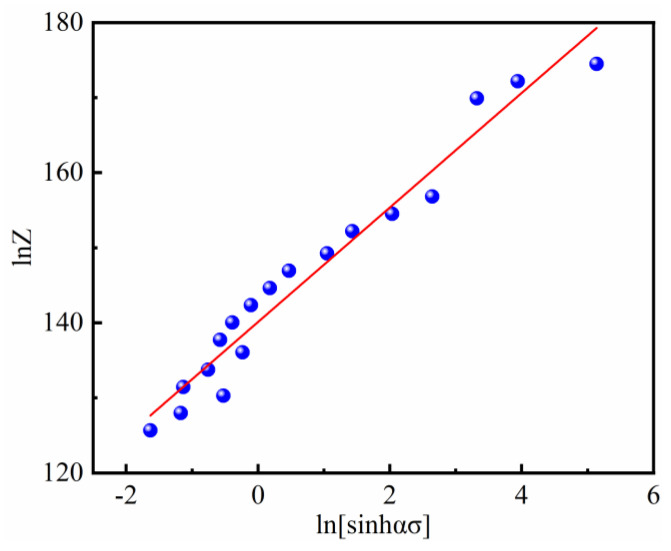
Relationship between the *Z* parameter and flow stress.

Substitute *Q* value and deformation conditions into Equation (4) to obtain the Z value. Then the lnZ-ln[sinh(*ασ*)] relationship diagram together with corresponding fitted linear fitting can be obtained, as shown in Figure 7. Obviously, the relationship exhibited a good linear correlation, thus demonstrating that the flow stress and strain behavior of SJTU-1 alloy at a high temperature can be well described by the *Z* parameter and the established equation is valid in the whole deformation. From Equation (13), the slope of the straight line in Figure 7 is the stress index n, and its intercept is lnA. From the fitting result, the stress index *n* is 7.6257, and from ln*A* = 140.10084, the material constant *A* = 6.999 × 10^60^ s^−1^ can be obtained finally.

As a result, Substituting material constants such as *A*, *Q*, *n* and *α* into Equation (3), the flow stress equation expressed by the Arrhenius relationship with a modified hyperbolic sine function of SJTU-1 alloy can be written as follows:

(14) $$\dot{\varepsilon }=6.999\times 10^{60}{\left[\sinh(0.00628\sigma )\right]}^{7.625}\exp[-1322786/(RT)]$$

Once substituted the material constants obtained above into Equation (5), the flow stress equation of SJTU-1 alloy expressed by *Z* parameter can be obtained:

(15) $$\begin{array}{c} \sigma =159.236\ln\{{\left(Z/6.999\times 10^{60}\right)}^{1/7.625}+\\ {\left[{\left(Z/6.999\times 10^{60}\right)}^{2/7.625}+1\right]}^{1/2}\} \end{array}$$

where $Z=\dot{\varepsilon }\exp\left(\frac{1322786}{RT}\right)$. The dependence of the peak stress on the *Z* parameter SJTU-1 alloy has been quantitatively described, providing theoretical guidance for thermal deformation.

### 3.3. Processing Map

#### 3.3.1. Processing Map Theory

The true stress-strain curve clearly expresses the relationship between the flow stress and the deformation parameters, and indirectly determines the variation of the microstructure, which is in favor of verifying the thermal deformation mechanism of the material. As an energy dissipation system during thermal processing, the material has the characteristics of dynamic, irreversible, and non-linear dissipation. The thermal processing map can optimize the material thermal process parameters and has better guidance on the evolution of the microstructure when thermally deformed under different conditions. It has been widely used in actual production. Moreover, the processing map can be used for evaluating the workability of the material under different deformation temperatures and strain rates. Generally, the dynamic materials model (DMM) [23] can be used to describe the deformation behavior of materials. The basic principle of the model is: firstly, the thermally deformed parts are regarded as an energy dissipator, then the process of plastic deformation, the processed part will consume the total energy (*P*) input from the outside in two ways: The first is the energy required for the plastic deformation of the part, denoted by *G*; The second is the energy consumed by the evolution of the microstructure during the deformation of the part, denoted by *J*. The total energy *P* can be expressed as:

(16) $$P=G+J=\int_{0}^{\dot{\varepsilon }}\sigma d\dot{\varepsilon }+\int_{0}^{\sigma }\dot{\varepsilon }d\sigma$$

The strain rate sensitivity index *m* of material under certain stress conditions can be expressed as:

(17) $$m=\frac{dJ}{dG}=\frac{\dot{d}d\sigma }{\sigma d\dot{\varepsilon }}={\left|\frac{\partial (\ln\sigma )}{\partial (\ln\dot{\varepsilon })}\right|}_{\varepsilon ,T}$$

Flow stress can be expressed as:

(18) $$\sigma =K{\dot{\varepsilon }}^{m}$$

Then the dissipation factor *J* can be expressed as:

(19) $$J=\int_{0}^{\sigma }\dot{\varepsilon }d\sigma =\int_{0}^{\sigma }{\left(\frac{\sigma }{K}\right)}^{\frac{1}{m}}d\sigma =\frac{m}{m+1}\sigma \dot{\varepsilon }$$

where 0 < *m* ≤ 1. When *m* = 1, the material is in an ideal dissipative state, and the dissipation factor *J* reaches its maximum value, i.e., *J* = *J*_max_ = $\sigma \dot{\varepsilon }/2$. For nonlinear dissipation, the power dissipation efficiency *η* can be used to reflect the power dissipation characteristics of the material, namely:

(20) $$\eta =\frac{J}{J_{\max}}=\frac{2m}{m+1}$$

The value of *η* determines the proportion of the energy dissipated in structural transformation. That means the larger *η* is, the better the workability is, which is of great guidance for material processing. The relationship of power dissipation efficiency *η* with deformation temperature and strain rate constitutes the power dissipation map of the material [24,25]. 

The stability of the material during hot deformation is a crucial performance as the instability tends to introduce the undesired occurrence of defects. Based on the maximum rate of entropy, the ξ parameter is used as a criterion for evaluating the flow instability of a material [26]. The instability phenomenon occurs when the strain rate exceeds the variation rate of entropy. The proposed ξ parameter is written as,

(21) $$\xi (\dot{\varepsilon })=\frac{\partial \ln\left(\frac{m}{m+1}\right)}{\partial \ln\dot{\varepsilon }}+m<0$$

where $\xi (\dot{\varepsilon })$ is a dimensionless instability parameter coupled with the deformation temperature and strain rate. The area with negative values on the energy dissipation diagram is the deformation instability area, which is called the deformation instability zone. Superimposing the instability map on the power dissipation map constitutes the thermal processing map of the material. According to the processing map, the rheological instability zone and safety zone of material processing can be determined. In the safe processing area of the material, the larger *η* is, the better the processability of the material is in this area.

#### 3.3.2. Establishment and Analysis of Hot Processing Map

**Figure 8:**
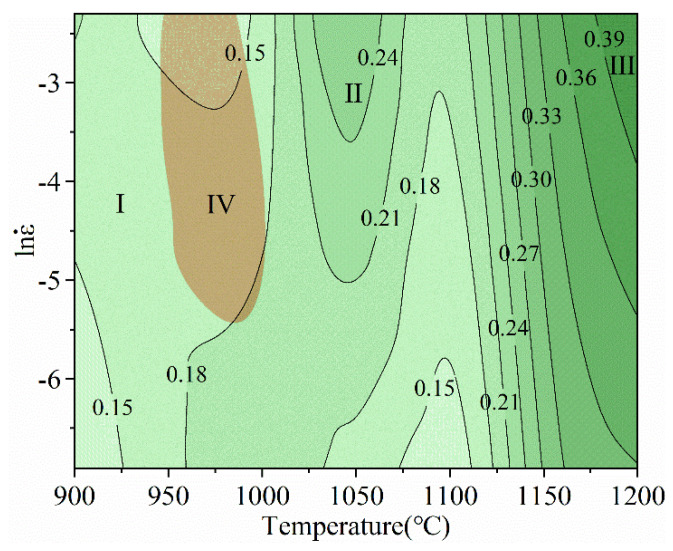
Processing map for SJTU-1 alloy at a strain of 0.5.

According to the abovementioned equations, the power dissipation map and the instability map are superimposed together to form a DMM processing map corresponding to a true strain of 0.5, as shown in Figure 8. The numbers on the contour line in the figure represent the power dissipation coefficient and the unit is %. It stands for thermal working performance, generally the bigger the value is, the better the performance are. From the low-temperature area to the high-temperature area, the power dissipation efficiency tends to increase. This change is related to the different high-temperature deformation mechanisms of the alloy. The increment of the deformation temperature makes the completely mutual destruction and recombination of the dislocations and promotes the nucleation and growth of the subcrystalline. Nucleus growth further leads to an increase in the dynamic recrystallization of the alloy.

It is generally believed that the higher the power dissipation coefficient, the better the thermal processing performance. The area has been divided into four parts. The shaded area (Part IV) in Figure 8 represents the instability area and is located in 950–1000 °C, 0.005–0.1 s^−1^, which means $\xi (\dot{\varepsilon })$ < 0. If the alloy undergoes a shaping reaction under the corresponding process parameters in the instability area, various defects that are unfavorable to the microstructure may appear. The next three areas are the safe zone (Part I, Part II, and Part III). In Part III, the maximum ***η*** is 39% and located in 1175–1200 °C, 0.03–0.1 s^−1^. However, it is not the optimum condition area for the temperature may exceed the mushy zone (The maximum eutectic temperature is 1165–1195 °C). The second-largest value of *η* is Part II with the maximum value of 24% located in 1000–1075 °C, 0.005–0.1 s^−1^, which indicates the optimum process conditions have been confirmed. Furthermore, for the region within the temperature of 1125–1200 °C and strain rate of 0.001–0.1 s^−1^, the power dissipation efficiency increases uniformly.

### 3.4. Microstructure and Deformation Mechanisms

**Figure 9:**
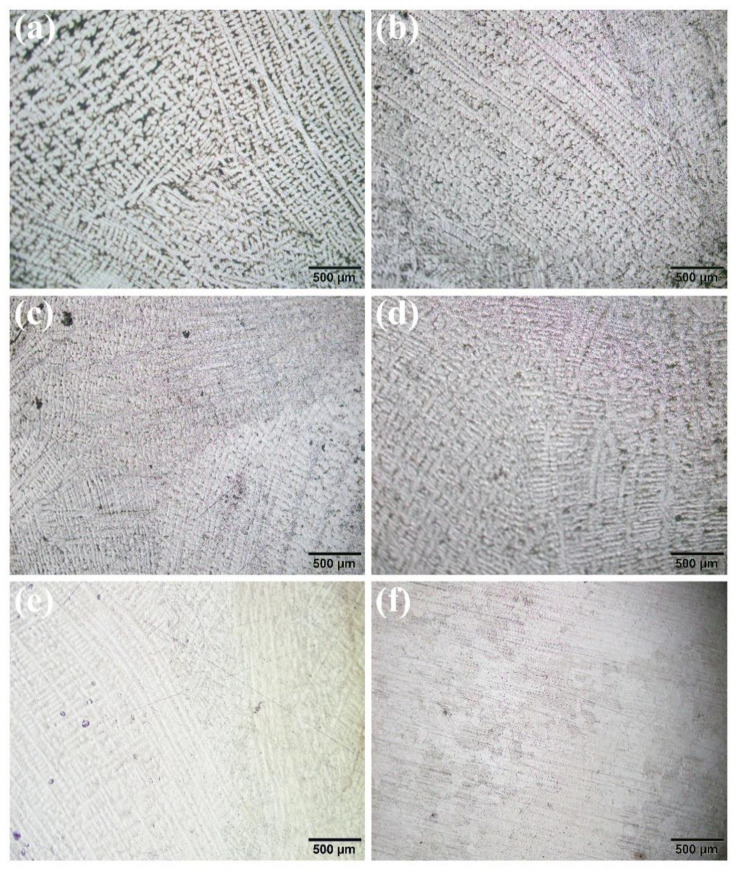
Optical microstructures of SJTU-1 alloy deformed with strain rate 0.001 s^−1^ at (**a**) 900 °C, (**b**) 1000 °C, (**c**) 1050 °C, (**d**) 1100 °C, (**e**) 1150 °C, and (**f**) 1200 °C.

Microstructure analysis of the hot compressed samples under different deformation conditions was carried out as can be seen from Figure 9. As is seen in Figure 9a, the coarse dendrites occur homogeneously in all specimens at low temperatures together with high strain rates. The structure is strongly refined compared to the initial specimen in Figure 1. The refinement can ascribe to the occurrence of dynamic recrystallization in the whole hot deformation [27]. As the temperature increased, the grain size decreased owing to the promotion of nucleation of dynamic recrystallization. From Figure 9d, the structure is quite different from the others. The reason is that the temperature is within the mushy zone, the alloy has transformed into a liquid, and the grain has to grow after the solidification, which makes a difference and does not present as a dendrite structure.

**Figure 10:**
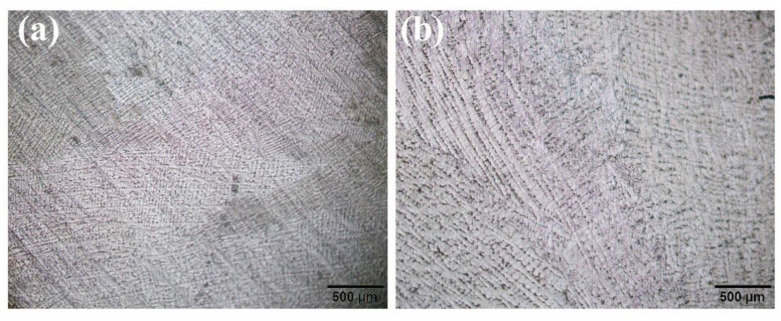
Optical microstructures of SJTU-1 alloy in the flow instability region at (**a**) 1000 °C, 0.1 s^−1^, (**b**) 1000 °C, 0.01 s^−1^.

Combined with Figure 8, it is concluded that the flow instability zone locates at the temperature of about 950–1000 °C and the strain rate of about 0.005–0.1 s^−1^. Figure 10 shows the microstructure of the flow instability zone at the condition of 1000 °C, 0.1 and 1000 °C, 0.01 s^−1^. As is seen, the dendritic structure has been torn into pieces with the trunk dismissed, which are harmful to the mechanical properties.

## 4. Conclusions

The hot deformation behaviors of high-throughput screened SJTU-1 alloy were investigated based on the flow curve, the constitutive equation analysis, processing map, and microstructural investigation. The following conclusions can be drawn:

(1) The high-throughput scanning is of great help for the acceleration of finding the alternative systems of potential superalloy from 22,398 component combinations. Based on such a method, a novel superalloy named SJTU-1 alloy has been obtained successfully.

(2) At the initial stage of the deformation. The flow stress moves fast to the peak value with the increased strain rates. At the same strain rates, the peak stress is decreased with the increased temperature. However, the peak stress shows the same tendency as the strain rates at the same temperature.

(3) The activation energy has been obtained: *Q* = 1322.786 kJ/mol. The deformation constitutive equation of SJTU-1 alloy under high temperature is described as follows: $\dot{\varepsilon }=6.999\times 10^{60}{\left[\sinh(0.00628\sigma )\right]}^{7.625}\exp[-1322786/(RT)]$. Furthermore, the material constants (i.e., *α*, *n* and *A*) in the Arrhenius constitutive equation have also been confirmed.

(4) The processing map of SJTU-1 alloy at different strain rates is established based on the power dissipation efficiency and instability parameter. Additionally, the instability zone has been defined thus in turn the optimum hot deformation condition was determined at the temperature range of 1000–1075 °C, and the strain rate range 0.005–0.1 s^−1^.

(5) The microstructure investigation indicates that at 900 °C the large, knobby dendrites emerge. However, the large dendrites have gradually been torn into a piece when the strain rate is 0.01 s^−1^ and 0.001 s^−1^ at the temperature of 1000 °C, indicating that the strain rate significantly governs the characteristics of the microstructure.

## Figures and Tables

**Table 1:** Composition of SJTU-1 alloy.

(wt.%)	Co	Cr	Al	Ti	Nb	W	Mo	Ni
	30.29	14.89	3.99	2.00	2.02	4.03	<0.001	Bal.

## Data Availability

Data sharing not applicable.
